# The preparation of a three dimensional terbium doped reduced graphene oxide aerogel with photoluminescence and paramagnetic properties†

**DOI:** 10.1039/c7ra12087g

**Published:** 2018-03-05

**Authors:** Keqin Chen, Hui Gao, Dongdong Wang, Xiaolong Li, Deying Wang, Waheed Ullah Khan

**Affiliations:** School of Physical Science and Technology, Key Laboratory for Magnetism and Magnetic Materials of Ministry of Education, Lanzhou University Lanzhou 730000 P. R. China hope@lzu.edu.cn +86-931-8913554 +86-18993181143; National & Local Joint Engineering Laboratory for Optical Conversion Materials and Technology, Key Laboratory of Special Function Materials and Structure Design, Ministry of Education P. R. China; Shanghai Synchrotron Radiation Facility, Shanghai Institute of Applied Physics, Chinese Academy of Sciences Shanghai 201204 P. R. China lixiaolong@sinap.ac.cn

## Abstract

A 3D reduced graphene oxide (rGO) material with good photoluminescence (PL) and magnetism properties was self-assembled using the hydrothermal method. The resultant material possessed a flower-like structure, which leads to a high surface area. To dehydrate the hydrogel for conservation, the sublimation process we use can effectively maintain the porosity as it uses the freeze drying method. Then, rGO was endowed with paramagnetism and green photoluminescence properties *via* the introduction of Tb ions, which also reinforces the significance of the building block. Finally, the coordination between Tb ions and the carbonyl was proven with photoluminescence excitation (PLE) spectra and the UV-vis absorption spectra, which are likely to offer an effective way to study the carbonyl compound.

## Abstract 2

A detailed mechanism for the ferromagnetism and photoluminescence of a composite, reduced graphene oxide doped with terbium ions (rGO/Tb), is proposed.

## Graphical abstract

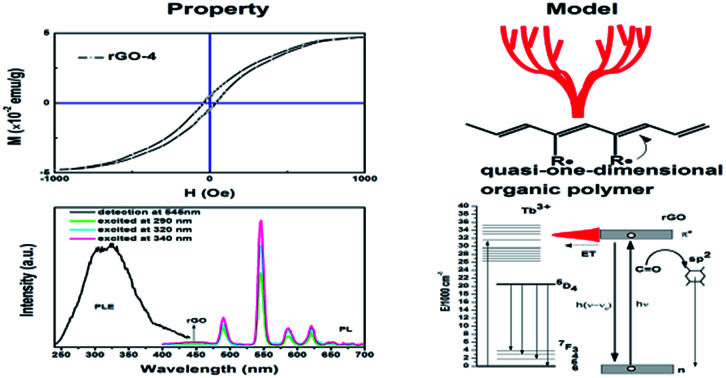

## 1. Introduction

Graphene has attracted great interest in research fields, due to its excellent electrical properties and high specific surface area.^1,2^ However, finely-sized graphene causes environmental pollution and suffers from poor integral mechanical properties while aggregated graphene monoliths lose the large surface area.^3,4^ Thus, it is necessary to integrate graphene sheets into 3D porous structures.^5–7^ So far, some work has made considerable progress in the synthesis of novel configurations, such as millispheres and plant stem structures.^3,5^ Another research hot topic focuses on the multi-functionalization of graphene, leading to the tremendous development of graphene and its derivatives, such as in electrodes, catalysis carriers, sensors and chemical filters.^8–11^ Graphene is also considered a promising spintronic device owing to its room-temperature spin transport and long spin diffusion lengths.^12^ However, pristine graphene is usually intrinsically diamagnetic. Introducing some foreign atoms or defects in the delocalized π bonding network of graphene is a widely utilized method in its practical application or in theoretical calculations to tailor its magnetism.^13,14^ And it is imperative to study the exact site where foreign ions are located, as not knowing this prevents us from understanding the system, and even the ion exchange process in lithium ion batteries and filter systems.

Our group has focused on rare earth ion doped materials, which possess good photoluminescence properties, high fluorescence quantum efficiencies, and low toxicities. The compounds (rGO/europium and rGO/Tb) had excellent luminescence properties and quenching abilities with rhodamine-B in our previous work.^15,16^ Meanwhile, terbium (Tb) plays a crucial role among the lanthanum elements, owing to its characteristic green emission and paramagnetism at room temperature. Thus, Tb can be exploited to tune magnetism and determine binding sites by measuring its optical behavior.

As a rich porous texture is prepared using the hydrothermal method, the dry method is a crucial link in the production chain, directly related to the mechanical strength and the surface area.^5^ So it is a challenge to clarify the link between the dry method and the pore properties. Besides, photoluminescence (PL) has become a technique used to study acceptor–donor recombination and the defects associated with trap levels.^10^ There are few studies on the binding sites of dopants in graphene using the PL method.

Herein, we report a 3D multifunctional cylinder, reduced graphene oxide doped with Tb ions, readily obtained by the hydrothermal method. Two main factors, the amount of water and use of the dry method, are studied in the 3D building process. The photoluminescence behavior of Tb ions and graphene indicates an energy-transfer process between them as do the binding sites of the Tb ions. It is noted that the introduction of Tb endows graphene with paramagnetism. In general, the photoluminescence and paramagnetic properties of the resultant composite are based on 3D modeling. Hence, this 3D structure and multifunctional material can potentially be applied in 3D devices and models.

## 2. Experimental

### 2.1 Materials

Tb_4_O_7_ (99.9%) was purchased from Shanghai Chemical Company. Potassium permanganate, hydrogen peroxide, potassium peroxodisulfate, phosphorus pentoxide, graphite powder, sodium nitrate, concentrated sulfuric acid and hydrochloric acid were all analytically pure. GO was synthesized using the modified Hummers method. Tb_4_O_7_ was dissolved in hydrochloric acid, and dried at 333 K for 24 h to obtain rGO-0, namely TbCl_3_.

### 2.2 Preparation of a series of rGO aerogels

A certain amount of TbCl_3_ aqueous solutions were dispersed into GO. Then, the dispersions were transferred into a Teflon tube and heated at 453 K for 8 h to obtain hydrogels. The different cylinder-like sponges were obtained after freeze-drying treatment. rGO-1 is made of 15 ml of purified water and 30 ml of GO (2 mg ml^−1^); rGO-2 is made of 15 ml of TbCl_3_ aqueous solution (0.3 mg ml^−1^) and 30 ml of GO (2 mg ml^−1^); rGO-3 is made of 2.5 ml of purified water and 20 ml of GO (3 mg ml^−1^); rGO-4 is made of 2.5 ml of TbCl_3_ aqueous solution (2 mg ml^−1^) and 20 ml of GO (3 mg ml^−1^); rGO-5 is made of 2.5 ml of TbCl_3_ aqueous solution (2 mg ml^−1^) and 20 ml of GO (3 mg ml^−1^) and exposed to air for 1 day before freeze-drying treatment.

**Fig. 1:**
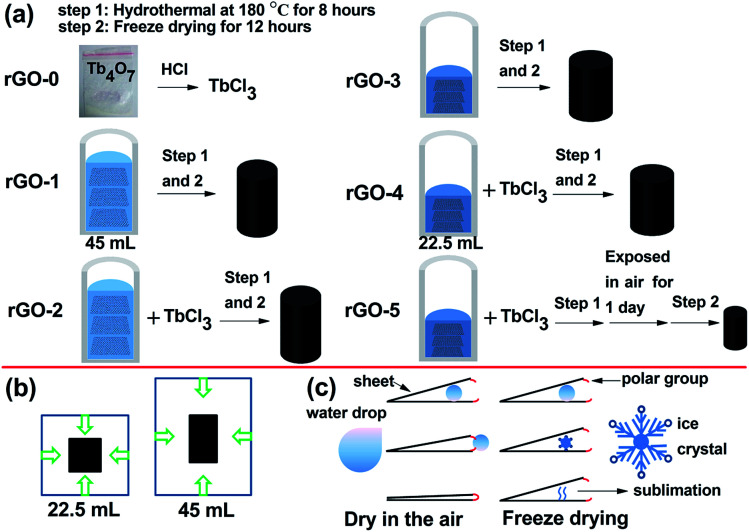
Drawings of the preparation procedure for the rGO series (a) and schematic diagrams for nucleation (b) and drying methods (c) of rGO.

Here, the liquid volume (22.5 ml and 45 ml) is a factor affecting the geometrical parameters and magnetism properties of rGO, as shown in Fig. 1.

### 2.3 Characterization

The sample’s morphology was examined using scanning electron microscopy (SEM, Hitachi, S-4800), powder X-ray diffraction (XRD) (Rigaku, Japan), X-ray photoelectron spectroscopy (XPS, PHI-5702, Physical Electronics), Raman spectroscopy (Renishaw InVia Raman microscope), Fourier transform infrared spectroscopy (Nicolet NEXUS 670 FTIR) and transmission electron microscopy (TEM, Tecnai G2, FEI Company). The PL spectra were obtained with an FLS-920T fluorescence spectrophotometer. The magnetization was characterized using a vibrating sample magnetometer (VSM). The thickness of rGO-2 was measured using atomic force microscopy (AFM) (Digital instruments Nanoscope III A).

## 3. Results and discussion

XPS measurement is a practical analytical approach to investigate the chemical composition and surface electronic state. The XPS spectrum shows the presence of carbon, oxygen and terbium atoms, as shown in Fig. S1a.† The C 1s peak is divided into three Gaussian peaks centered at 284.5, 285.5 and 287.8 eV, suggesting different energies of carbon. The binding energies 284.5 eV, 285.5 eV and 287.7 eV match the present sp^2^ hybridized C atoms (C=C), the bond between C and O (C–O), and the carbonyl (C=O), respectively. The O 1s core level spectrum shown in Fig. S1b† contains two peaks, centered at 530.8 eV and 533.1 eV. It has been reported that the binding energy of the O–H bond in the hydroxyl group (O–H) should be 529.0–531.1 eV, while it is approximately 533.0 eV for C–OH, C=O, and O=C–OH. The binding energy related to Tb is clarified in Fig. S1c and d.† In these spectra, the XPS signals resulting from Tb 3d (3d_5/2_ and 3d_3/2_) orbitals are intricate, including some splitting peaks around the characteristic peaks of the Tb 3d region, centered at 1242.1 eV and 1275.8 eV. The separated doublets for Tb^3+^ (1239.1 eV and 1274.9 eV) and Tb^4+^ (1242.2 eV and 1279.8 eV) are derived from the hybridization of the Tb 4f orbital with the host orbital and fractional occupancy of the valence 4f orbital. The relatively intense peaks at 1247.1 eV and 1276.1 eV are assigned to the 3d_5/2_ and 3d_3/2_ orbitals of Tb^3+^, respectively. From the Tb 4d core level spectrum (Fig. S2†), three regions are observed for this system, suggesting multiplet splitting in the 4d band. The line in the range 148–151 eV is due to Tb^3+^, those in the range 156–165 eV arise from Tb^4+^ while the mid part is likely to be related to complex formation between Tb, O, and C. Finally, it is obvious that Tb has been introduced into the composite and the calculated content of Tb is about 1%, as shown in Fig. S1.†

A Raman spectrum of the product is shown in Fig. S3a.† The D peak (1345 cm^−1^), G peak (1590 cm^−1^) and 2D peak (2690 cm^−1^), used to describe the quality of graphene, indicate that the as-prepared material is stacked by graphene sheets. Fig. S3c† shows the XRD spectrum of rGO-2; the diffraction peak appearing at about 20 degrees originates from the lattice plane (002) while the position of GO is at 10 degrees. This result supports the fact that GO has been changed into rGO (the interplanar spacing decreases from 8–9 nm to 4–5 nm). Some typical molecular vibration patterns of the material are observed in the FTIR spectra (Fig. S3a†). Going from high to low wavenumber, there is a region at 3438 cm^−1^, attributed to the O–H stretching vibration, the weak 1713 cm^−1^ peak corresponding to HO–C=O, and the 1560 cm^−1^ peak due to unoxidized sp^2^ C=C, as well as the 1155 cm^−1^ C–O vibration peak. The TEM image of rGO-2 is shown in Fig. S3d.† It can be seen that the composite is stacked by sheets and that the thickness is about 5.1 nm (Fig. S7†) on the margin, namely that it is thick in the middle and thin in both sides. All results suggest that the composite is mainly constituted of rGO and that Tb ions have already been doped in the composite.

**Fig. 2:**
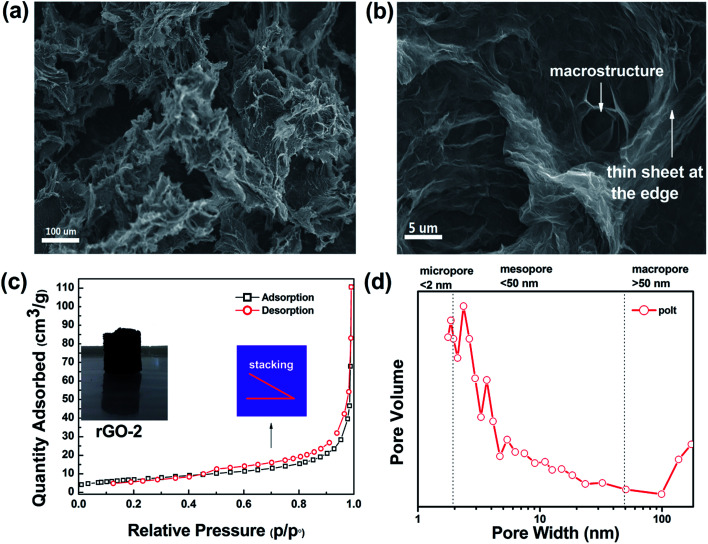
The different resolution SEM images at (a) 100 μm and (b) 5 μm, BET spectrum (c) and the size distribution (d) of rGO-2.

Commonly, the microstructure is related to the volume. We obtained rGO-3 and rGO-4 by water regulation (reducing the volume of water), as shown in Fig. 1a. It is important to note that the dehydration and skeleton collapse happen in the air for these hydrogels with elapsing time; rGO-5 is obtained due to this effect (Fig. S4†). Table 1 shows the monolith volumes and BET data for rGO-2, rGO-4 and rGO-5. Surprisingly, the mesoporous volumes and monolith volumes do not match. As Fig. 1b depicts, the monolith volume is inclined to relate to the nucleation area while the mesoporous volume is related to dehydration progress. To better understand the reduction of surface area by dehydration, we studied the pore structure and morphology of rGO-2. The N_2_-sorption isotherm of rGO-2 is fitted in Fig. 2c, and is categorized as Type 3 according to IUPAC classifications of mesoporosity (from 2 nm up to 50 nm) and macroporosity (exceeding 50 nm). This result agrees with the size distribution, as shown in Fig. 2d, suggesting that mesoporosity is formed by stacking. In Fig. 2a and b, the material displays randomly distributed thick sheet-like petals at the micro level, forming a coralline structure with many channels. After magnifying the picture 20 times, finer and dense vortex-like macropores can be observed and the sheets seem thinner at the edge of the extension, which produces greater interlayer spacing. Due to having a smaller nucleation area, more sheets of rGO-4 take part in stacking compared with those in rGO-2, resulting in an enhancement in surface area. As shown in Fig. 1c, in the atmospheric evaporation period, the sheets of rGO-5 are pulled together by their affinity for the droplet, leading to the annihilation of mesoporosity and collapse of the skeleton. However, the shape and the structure of rGO-4 change imperceptibly in the freeze drying, due to the phenomenon of ice’s rapid sublimation.

**Table 1:** The geometric parameters and magnetic susceptibilities of rGO 0–5

	rGO-0	rGO-1	rGO-2	rGO-3	rGO-4	rGO-5
Monolith volume^a^ (10^−6^ m^3^)		3.18	2.83	2.45	2.26	0.9
Specific surface area (m^2^ g^−1^)			25.3		154.0678	0.2048
*χ* a (10^−7^)	732	−4.54	7.45	−29.5	26.6^a^	14.6

a The magnetic susceptibility at the end of the loop.

**Fig. 3:**
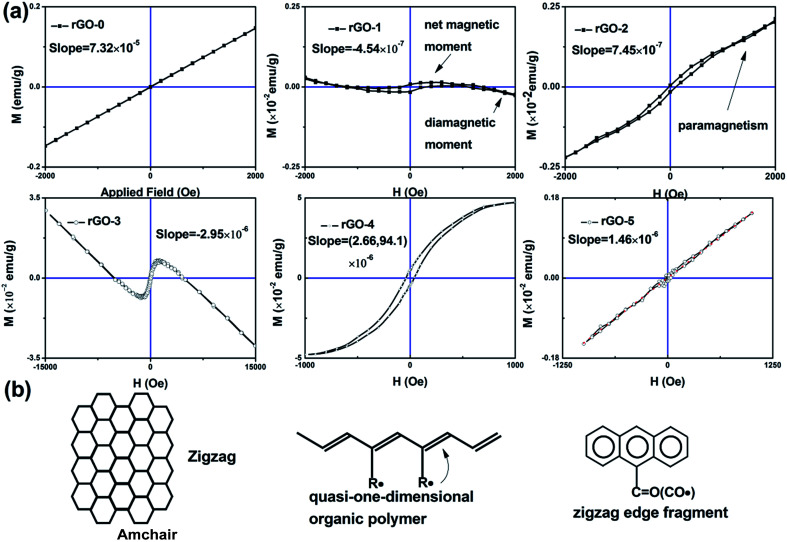
The hysteresis loops of the rGO series (a) and the possible models of rGO with magnetism (b).

The vibrating sample magnetometer was used to investigate the magnetism of the composite, as shown in Fig. 3. It is obvious that there is paramagnetism in rGO-0 due to its Curie point being below room temperature. There are two states in rGO-1: the weak paramagnetism at the beginning stage and the diamagnetism during the later magnetic field stage, which is attributed to the integrated contribution from the net magnetic moment and diamagnetic moment. Meanwhile the diamagnetic signal faded away when Tb ions were added, as shown in rGO-2. Compared with rGO-0, rGO-1 and rGO-2 both have a hysteresis loop, indicating that the hysteresis originates from rGO. To study the intrinsic magnetism of rGO and the role of Tb ions in the composite, we measured the magnetism of rGO-3, rGO-4 and rGO-5 and calculated the magnetic susceptibility (*χ*), as shown in Table 1. It was found that rGO-1 and rGO-3 have the same shape except for *χ*, as do rGO-2 and rGO-4, while there is no loop in rGO-5. To understand the phenomena, we refer to the well-known organic polymer model of a quasi-one-dimensional organic polymer, in which it is believed that the magnetism originates from the strong coupling between the radical and the C=C bond.^17^ Combined with the BET data, it was found that the porosity and the sp^2^ structure are critical for the loops in the bulk material. This may be explained by stacking from the van der Waals bond in the *z* direction, destroying the coupling with unpaired electrons. It was also found that paramagnetism appears in all the composites doped with Tb ions, suggesting the regulating effect of the Tb ions in magnetism. So we successfully integrated paramagnetism into rGO.

**Fig. 4:**
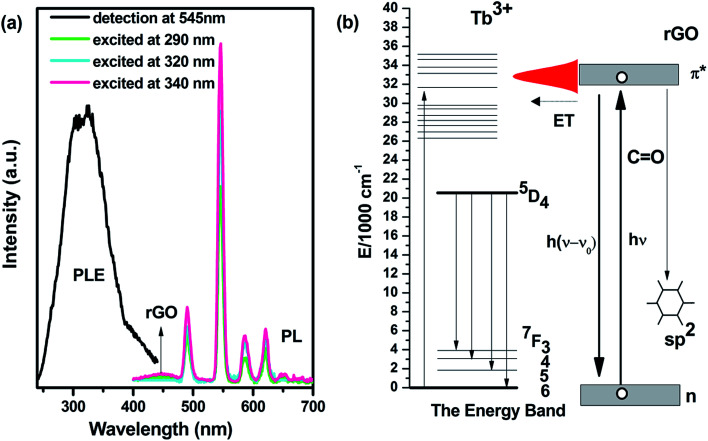
(a) Photoluminescence excitation and emission spectra of rGO-2. (b) The diagrammatic drawing of the possible transitions during photoluminescence of rGO-2.

Another enhancement for rGO-2 is the luminescence compared with that of rGO-1. There is no light emission for rGO-1 in the visible range. In contrast, rGO-2 has excellent photoluminescence (PL) and wide photoluminescence excitation (PLE), as shown in Fig. 4a. It is obvious that rGO-2 has narrow emissions centered at 489, 544, 585 and 621 nm, as well as the weak wide band ranging from 400–480 nm, leading to intense green luminescence with weak blue light. These originated from the ^5^D_4_–^7^F_*J*_ (*J* = 3, 4, 5, 6) transitions of Tb ions and the sp^2^ structure of rGO, respectively.^18,19^ And the PLE spectra (monitored at 544 nm, detailed in Fig. S5†) show a broad excitation region ranging from 250–400 nm, which is different from rGO-0 which is mainly excited in discrete peaks.^16^ The band gap of graphene measured by UV-vis absorption spectroscopy was used to confirm the variation in PLE. In Fig. S6,† a widening peak appears at about 300 nm from rGO-1, which is assigned to the n–π* transition of C=O. At the same time, Tb ions tend to bond with the oxygen functional group. As Fig. 4b shows, there is probably energy transfer between Tb ions and rGO-2. Meanwhile, another luminescence center of rGO should be ascribed to the contribution of the local sp^2^ structure, which is more like carbon dots. These results suggest that the additional functionality has been endowed to our composite and Tb ions locate near to the C=O group.

## 4. Conclusion

In summary, a material, reduced graphene oxide doped with Tb ions, was synthesized simply, and it is three dimensional, has a mesoporous structure, and possesses paramagnetism and photoluminescence properties. By introducing Tb ions into rGO, the material was endowed with paramagnetism and photoluminescence properties, suggesting its potential application in controllable synthesis and optical detection fields. Furthermore, we demonstrated energy transfer between Tb ions and rGO, as well as locating the binding site of Tb ions in rGO, which provides the potential to extend to other rare earth compounds.

## Conflicts of interest

There are no conflicts to declare.

## Supplementary Material

RA-008-C7RA12087G-s001
