# Supplementary material for: The preparation of a three dimensional terbium doped reduced graphene oxide aerogel with photoluminescence and paramagnetic properties
Source: RSC Adv. 2018 Mar 5;8(17):9287–92. doi: 10.1039/c7ra12087g (PMC9078696; doi:10.1039/c7ra12087g)
Supplement: RA-008-C7RA12087G-s001 [file RA-008-C7RA12087G-s001.pdf]

Electronic Supplementary Material (ESI) for RSC Advances.
This journal is © The Royal Society of Chemistry 2018

The preparation of a three dimensional terbium doped reduced graphene oxide aerogel with photoluminescence, paramagnetic properties

Keqin Chen[a‡], Hui Gao[a*‡], Dongdong Wang[a], Xiaolong Li[b] *, Deying Wang[a], Waheed Ullah Khan[a]

[a]School of Physical Science and Technology, Key Laboratory for Magnetism and Magnetic Materials of Ministry of Education, Lanzhou University, Lanzhou 730000, P.R. China, National & Local Joint Engineering Laboratory for Optical Conversion Materials and Technology, Key Laboratory of Special Function Materials and Structure Design, Ministry of Education. E-mail: hope@lzu.edu.cn; Fax: +86-931-8913554; Tel: +86-18993181143.

[b] Shanghai Synchrotron Radiation Facility, Shanghai Institute of Applied Physics, Chinese Academy of Sciences, Shanghai 201204, P. R. China. E-mail: lixiaolong@sinap.ac.cn

‡ These authors contributed equally to the article.

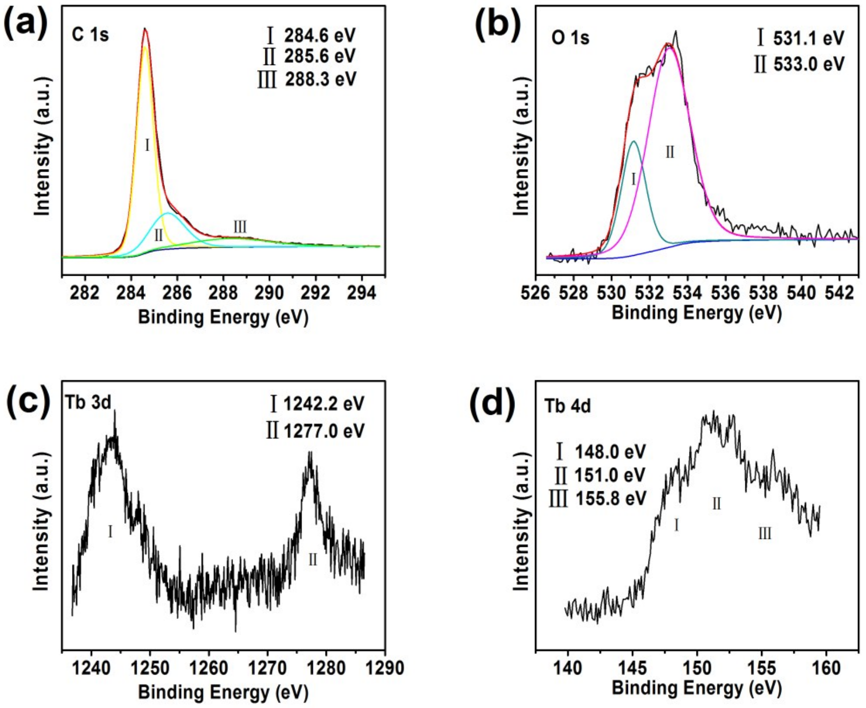

Fig. S1 the XPS survey spectrum of rGO-2. XPS high resolution spectra of C 1s (a), O 1s (b), Tb 3d (c), Tb 4d (d) core levels in rGO-2.

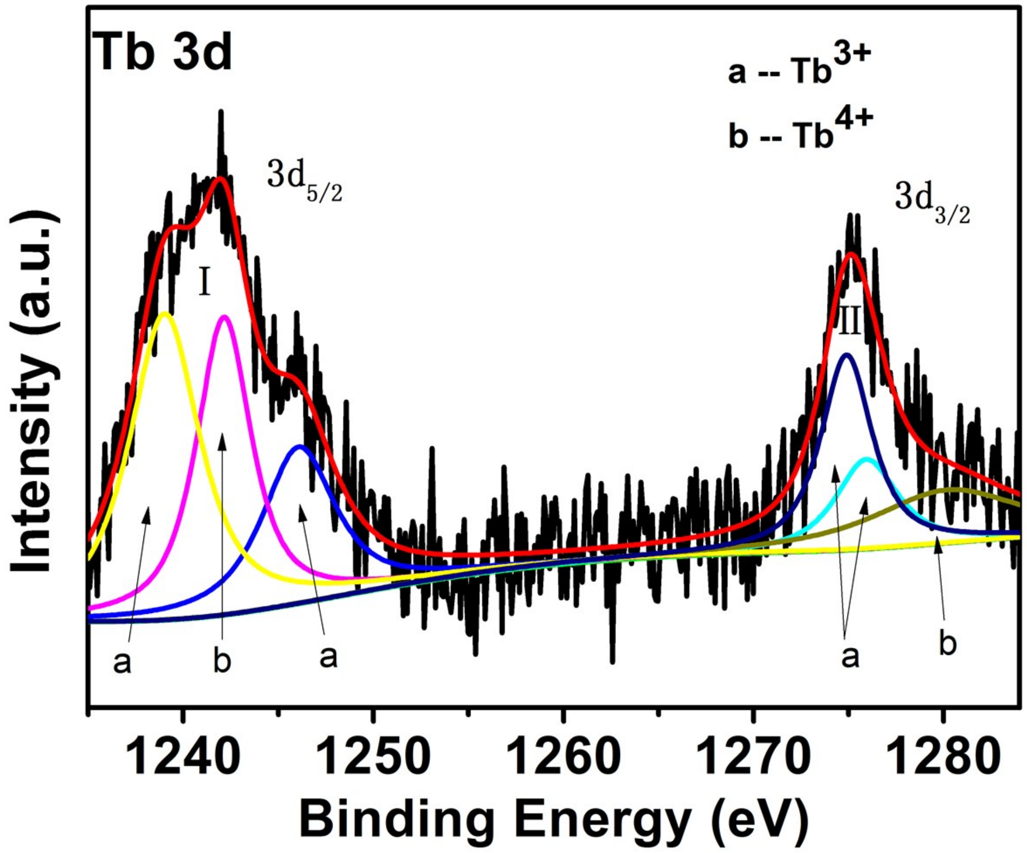

Fig. S2 the fitting curve of XPS spectrum for terbium atom.

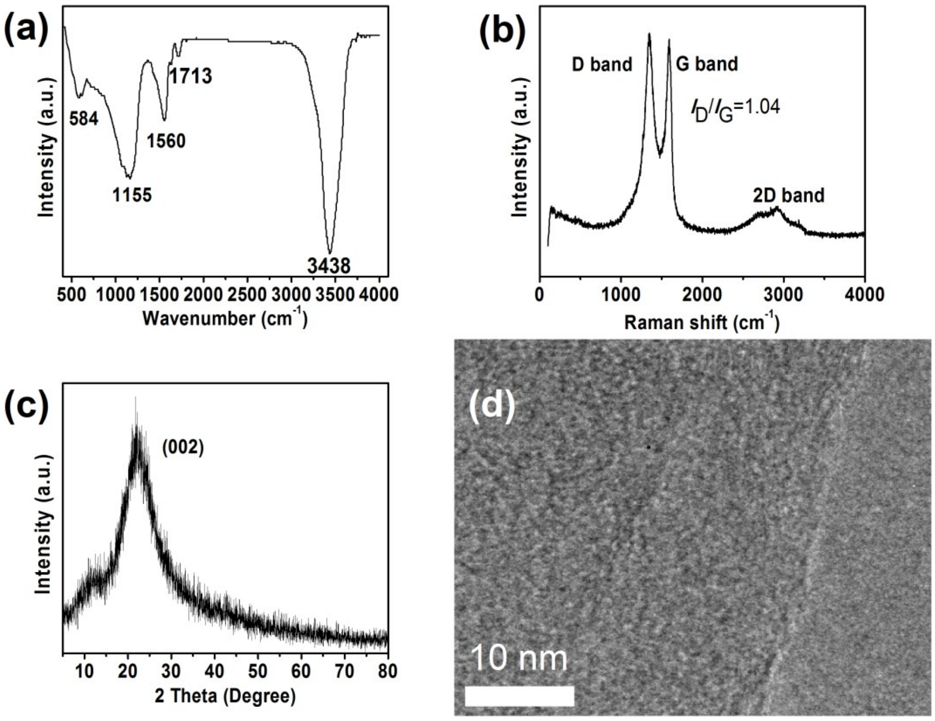

Fig. S3 FT-IR (a), Raman (b), XRD spectrum (c) and HR-TEM (d) of rGO-2.

Table S1 the BET data of the rGO-2

**Method**	**Surface Area**	**Pore Volume**	**Pore Size**
**BJH adsorption**	25.26 m^2/g	0.1701 cm^3/g	26.93 nm
**BJH desorption**	33.30 m^2/g	0.1713 cm^3/g	20.58 nm
	25.3 m^2/g (BET Surface Area)		9.68 nm (adsorption average pore width)

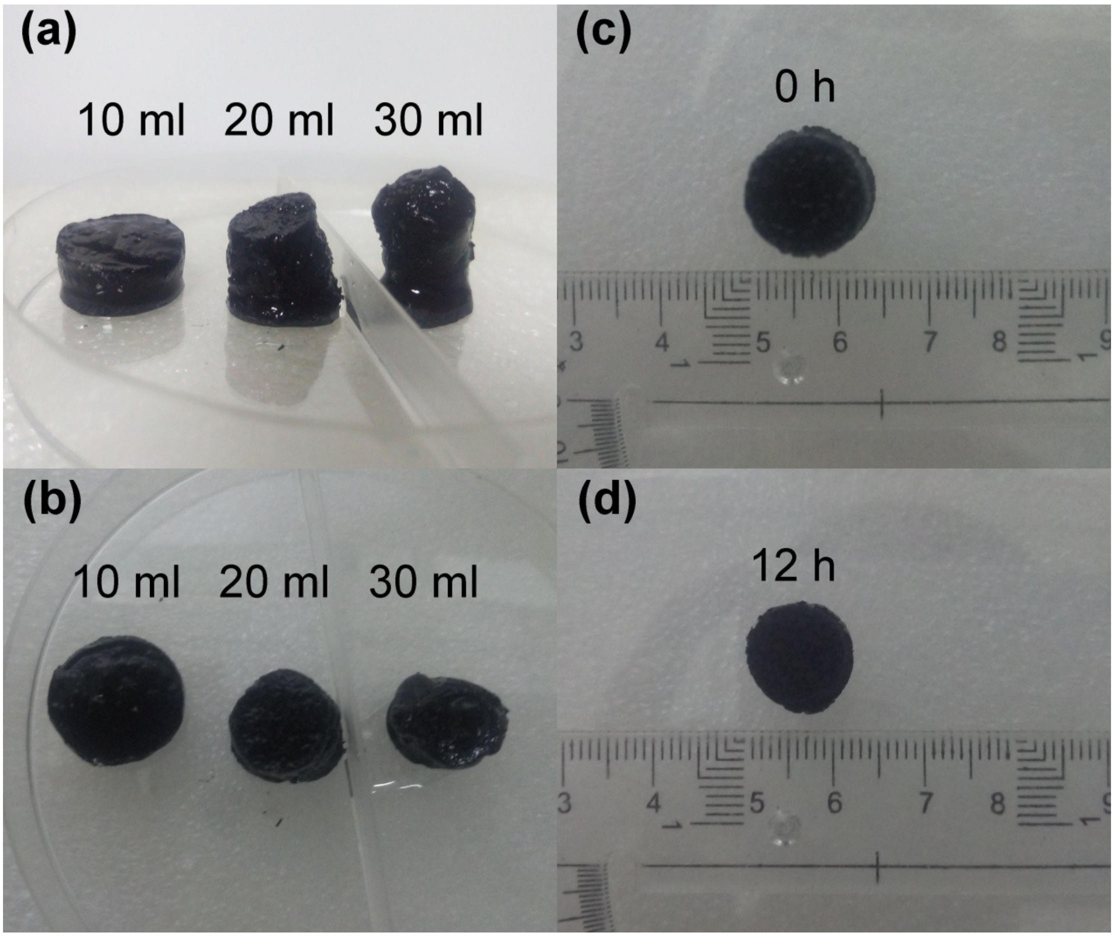

Fig. S4 the photos of nucleation area factor (a) and (b), air drying experiment (c) and (d) for volume of rGO.

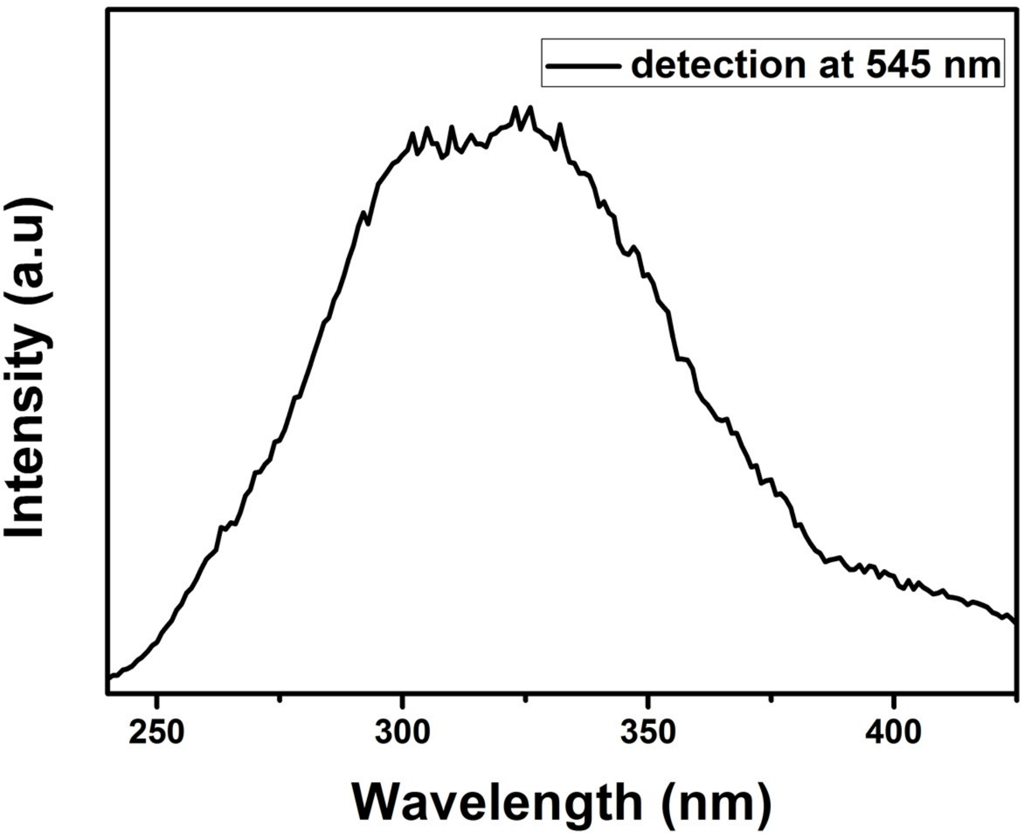

Fig. S5 PLE spectrum of rGO-2.

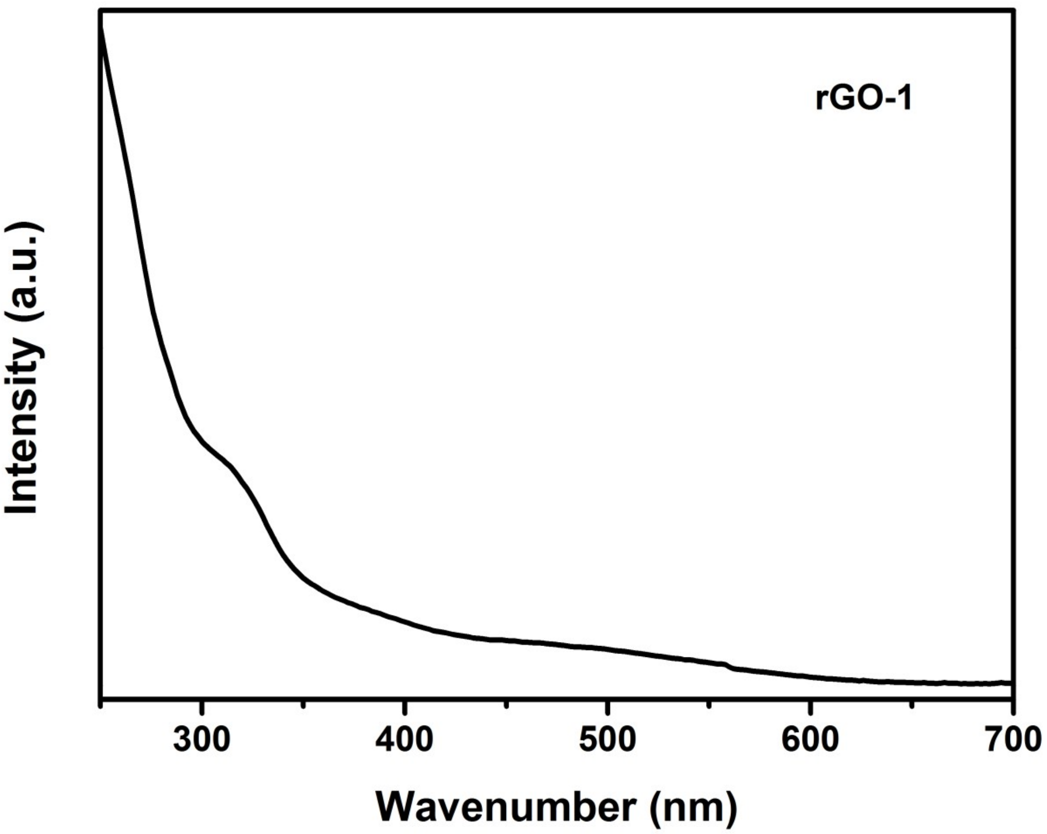

Fig. S6 UV-vis Adsorption spectrum of rGO-1.

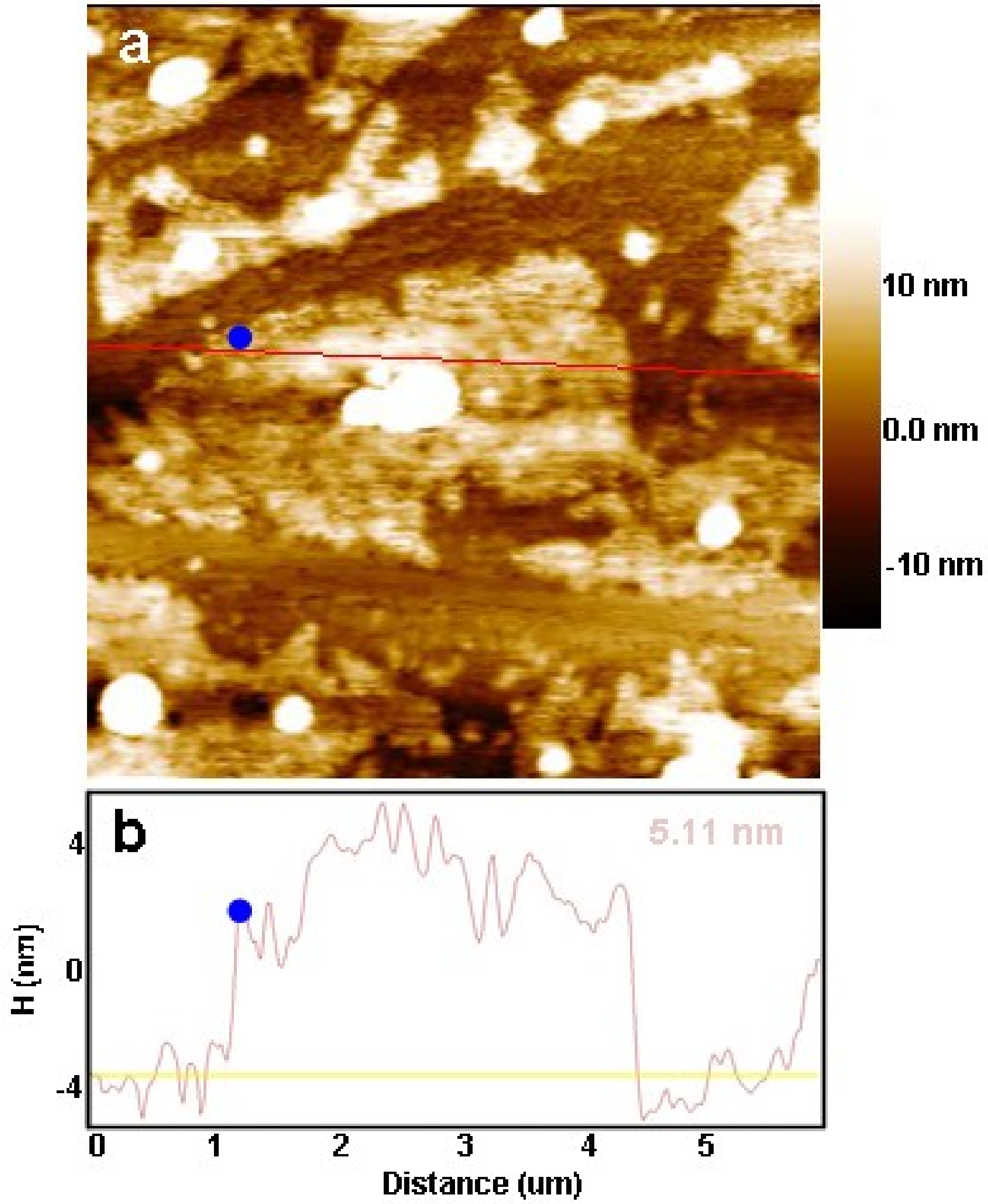

Fig. S7 (a) AFM image of rGO-2. (b) The height distribution according to the scanning line marked in the AFM image.
